# Reducing the number of catheter-associated urinary tract infections at an inpatient rehabilitation facility: A quality improvement project

**DOI:** 10.1017/ice.2023.97

**Published:** 2023-11

**Authors:** Clayton R. Walker, Jerry Jacob

**Affiliations:** 1 Department of Physical Medicine and Rehabilitation, University of Pennsylvania–Perelman School of Medicine, Philadelphia, Pennsylvania; 2 Division of Infectious Diseases, University of Pennsylvania–Perelman School of Medicine, Philadelphia, Pennsylvania

## Abstract

During the coronavirus disease 2019 (COVID-19) pandemic, the incidence of healthcare-associated infections (HAIs), including catheter-associated urinary tract infections (CAUTIs), increased significantly across the country. This report describes a quality improvement project aimed at reducing the incidence of CAUTIs at an inpatient rehabilitation facility.

Catheter-associated urinary tract infections (CAUTIs) are defined by the National Healthcare Safety Network (NHSN) as urinary tract infections (UTIs) that occur >2 days after insertion and <2 days after removal of an indwelling urinary catheter (IUC).^1^ During the coronavirus disease 2019 (COVID-19) pandemic, the incidence of healthcare-associated infections (HAIs) increased significantly across the country. Specifically, CAUTIs increased by 36% from 2019 to 2020.^2^ We experienced similar trends at our inpatient rehabilitation facility, a 58-bed standalone facility associated with an academic quaternary-care center. One CAUTI was identified in fiscal year (FY; July–June) 2019, 2 CAUTIs were identified in FY2020, and 4 CAUTIs were identified in FY2021. Root-cause analyses of the 4 cases from FY2021 revealed a few commonalities: (1) all 4 patients were admitted with an IUC, (2) none had a void trial done prior to symptom onset, and (3) all 4 were discharged without needing an IUC. Accordingly, we undertook a quality improvement project with the goal of initiating void trials earlier, thereby reducing the number of CAUTIs by 50% over a 12-month period.

## Methods

**Fig. 1.**
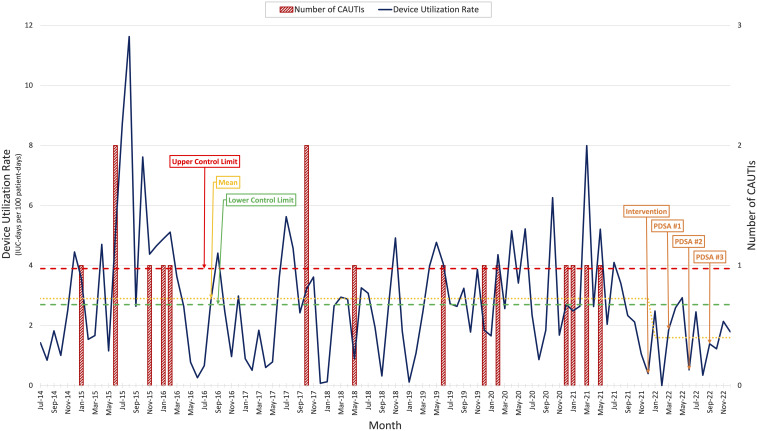
The chart used to monitor the success of the intervention. The upper control limit was set at 3.99 IUC-days per 100 patient days. The Lower control limit was set at 2.73 IUC days per 100 patient days. Mean is calculated for preintervention months and postintervention months. Note. CAUTIs, catheter-associated urinary tract infections; IUC, indwelling urinary catheter; PDSA, plan–do–study–act.

The number of CAUTIs per year was used as an outcome measure, and the device utilization rate was used as a process measure, which is reported as number of IUC days per 100 patient days. To establish normative values for both metrics, the available historical data were reviewed, which amounted to 7 years (137,051 patient days). The investigation revealed a historical CAUTI rate of 2 CAUTIs per year (1.67 CAUTIs per 10,000 patient days) and a historical device utilization rate of 2.94. In FY2021, the CAUTI rate was 4 CAUTIs per year (2.15 CAUTIs per 10,000 patient days) and the device utilization rate was 3.21. The average device utilization rate was 3.99 for months in which a CAUTI was identified, and 2.73 for months in which no CAUTI was identified. We used these values as upper and lower control limits respectively on a chart (Fig. 1), which was updated and reviewed monthly. To ensure that the intervention was not leading to patient harm, all patients who required transfer to an acute-care hospital were logged and reviewed for complications relating to IUC management.

A quality improvement (QI) team was formed with members from key stakeholder groups. Overall, 10 clinical staff were interviewed; 9 interviewees identified the lack of a standardized process for IUC removal as a significant barrier. Accordingly, the QI team created a standardized protocol outlining criteria to initiate a void trial, with IUC removal occurring on hospital day 1 (Supplementary Fig. 1 online). The protocol was initially designed as a “nurse-driven protocol,” allowing nurses the autonomy to remove an IUC without a physician order, thereby streamlining the process. Members of the QI team attended nursing staff meetings for 2 consecutive weeks and answered questions pertaining to the protocol. Additionally, the protocol was presented to clinicians, and printed versions were placed in all clinical areas. Patients with spinal cord injuries were initially excluded from the protocol due to the intricacies of managing a neurogenic bladder.

After implementation, plan–do–study–act (PDSA) cycles were planned quarterly or sooner if the device utilization rate trended above the upper control limit. In PDSA cycle 1, after implementation of the protocol, the device utilization rate decreased and remained below the upper control limit throughout the first quarter, with zero reported CAUTIs. After interviewing stakeholders, it became apparent that having a nurse-driven protocol was inefficient because providers were still being contacted for an order prior to removal of the IUC. As a result of this feedback, the protocol was modified to be physician driven. Although the protocol was not being executed exactly as planned, it still appeared to be successful at reducing the device utilization rate. This improvement prompted the QI team to expand the scope of the project to include patients with spinal cord injuries.

In PDSA cycle 2, stakeholder interviews revealed that providers were forgetting to use the protocol on admission, which was leading to minor delays in initiating the void trial. To address this problem, the protocol was embedded in the standard history and physical (H&P) note template that is used for all admissions. This change forced providers to interact with it on the day of admission.

In PDSA cycle 3, the feedback from stakeholders was positive, and the protocol was being effectively used with most patients who met the inclusion criteria. The decision was made to discontinue scheduled PDSA cycles and to reconvene only if the device utilization rate trended above the upper control limit.

All data were analyzed using Microsoft Excel (Microsoft, Redmond, WA). Comparisons were done using *t* tests when able. When data were heavily weighted with zero, no *P* values were calculated. *P <* .05 was considered statistically significant.

## Results

In the 12-month period following the implementation of the protocol (January 2022–December 2022), the device utilization rate decreased to 1.64 from 3.21 in FY2021 (*P* = .03), reflecting a 49% reduction (Fig. 1). This rate was also significantly lower than the historical average of 2.94 (*P* < .001). The CAUTI rate decreased from 4 CAUTIs per year (2.15 CAUTIs per 10,000 patient days) in FY2021 and a historical average of 2 CAUTIs per year (1.17 CAUTIs per 10,000 patient days) to 0 CAUTIs per year (Table 1). No adverse events were documented.

**Table 1.** Preintervention Data Compared to Postintervention Data

Variable	FY2021 (July 2020–June 2021)	Historical Average (July 2014–December 2021)	Postintervention (January 2022–December 2022)	% Change from FY2021 (*P* Value)	% Change from Historical Average (*P* Value)
Device utilization rate	3.21	2.93	1.65	49 (.03)	44 (<.001)
No. of CAUTIs per year (No. of CAUTIs per 10,000 patient days)	4 (6.71)	2 (3.97)	0	…	…

Note. CAUTI, catheter-associated urinary tract infection; FY, fiscal year; IUC, indwelling urinary catheter.

## Discussion

Implementing a standardized protocol outlining when to initiate void trials was associated with a significant reduction in device utilization rate and a CAUTI rate of zero. The case-mix index at the inpatient rehabilitation facility remained relatively stable from FY2019 to FY2022 (1.31, 1.38, 1.35, 1.33, respectively), and no other changes in product or policies regarding CAUTI prevention would have confounded this result.

This project highlights the importance of tracking both outcome and process measures in small facilities such as inpatient rehabilitation facilities. If only the number of CAUTIs had been tracked, it would have been impossible to recognize whether protocol changes had an impact on a month-to-month basis because this metric is heavily weighted to zero. Using the device utilization rate as a process measure made it possible to visualize the impact of protocol changes monthly, allowing for an iterative design process. This project also highlights the value of having scheduled PDSA cycles. If only the chart was used to trigger a PDSA cycle, no changes would have been performed through all 12 months of the project and the first iteration of the protocol would have been implemented despite being poorly adopted by staff.

This study had several limitations. It was conducted at a single-center inpatient rehabilitation facility, which may limit its generalizability to other inpatient rehabilitation facilities or to other clinical settings such as an acute-care hospital, long-term acute-care hospital, or skilled nursing facility. Also, there was no direct tracking of when or how frequently the protocol was used. The QI team instead relied on stakeholder interviews and a process measure to assess its effectiveness.

In conclusion, the QI team successfully examined the current practices of managing patients with IUCs, identified inefficiencies in the process, and applied an evidence-based countermeasure to reduce the number of CAUTIs. To sustain the improvement, the protocol (Supplementary Fig. 1 online) has been embedded in the standard H&P note template and the QI team plans to reconvene should the device utilization rate trend above the upper control limit.

## Supplementary material

For supplementary material accompanying this paper visit https://doi.org/10.1017/ice.2023.97. click here to view supplementary material
